# Associations between Diurnal 24-Hour Rhythm in Ambulatory Heart Rate Variability and the Timing and Amount of Meals during the Day Shift in Rotating Shift Workers

**DOI:** 10.1371/journal.pone.0106643

**Published:** 2014-09-11

**Authors:** Takahiro Yoshizaki, Toru Midorikawa, Kohe Hasegawa, Takeshi Mitani, Taiki Komatsu, Fumiharu Togo

**Affiliations:** 1 Graduate School of Agriculture, Tokyo University of Agriculture, Tokyo, Japan; 2 Faculty of Food and Nutritional Sciences, Toyo University, Tokyo, Japan; 3 Health Care Facility at Medical Corporation of Doaikai, Chiba, Japan; 4 School of Health Science, Department of Physical Therapy, Tokyo University of Technology, Tokyo, Japan; 5 Educational Physiology Laboratory, Graduate School of Education, The University of Tokyo, Tokyo, Japan; University of Alabama at Birmingham, United States of America

## Abstract

It has not hitherto been clarified whether there is an association between dietary behavior and circadian variation in autonomic nervous system activity among shift workers. This study examines diurnal 24-h rhythm in heart rate variability (HRV) and dietary behavior among rotating shift workers, while taking into account the sleep-wake cycle and physical activity. The subjects were 11 female and 2 male nurses or caregivers working in a rotating 2-shift system at a health care facility. All the subjects were asked to undergo 24-h electrocardiograph and step count recordings, and to record the time of each meal and the amounts of each food and beverage consumed. Coarse graining spectral analysis was used for approximately 10-min segments of HRV to derive the total power (TOT: >0.04 Hz) of the periodic components and the integrated power of periodic components in the low-frequency (LF: 0.04–0.15 Hz) and high-frequency (HF: >0.15 Hz) ranges. Then the ratio of HF power to TOT (HF nu) and the ratio of LF power to HF power (LF/HF) were calculated to assess cardiac vagal tone and cardiac sympathovagal balance, respectively. Single cosinor analysis was used to obtain 24-h period variations in both variables of HRV. Acrophases of HF nu and LF/HF expressed in time since awakening were significantly (p<0.05) delayed for subjects having breakfast at a later time after awakening. Multivariable regression analysis indicated that the timing of breakfast, the ratio of energy intake at dinner to total energy intake, and total energy intake were correlated to the acrophases of HF nu and/or LF/HF. These results suggest that the phase angle between circadian variation in cardiac autonomic nervous system activity and the sleep-wake cycle may be associated with dietary behavior in shift workers.

## Introduction

Shift workers have higher risks of health problems such as cardiovascular disease, abnormal metabolism, obesity, and cancer compared with day workers [1]–[6], while there is an increase in the social demand for them in an aging society. Misalignment of the circadian clock in the suprachiasmatic nucleus (SCN) with the sleep-wake cycle and other peripheral oscillators (i.e., circadian misalignment) has been indicated to be one of the causes of these health problems [7]–[10]. The misalignment has been observed during a simulated shift in the sleep-wake cycle [7], [11], [12]. In addition, in real-life shift work situations, our recent study has revealed that shift workers have a significant phase delay in cardiac autonomic nervous system activity compared to fixed day workers during the day shift [13].

Recent animal studies have suggested that the feeding schedule contributed to the modification of the circadian clock independently of the light-dark cycle [14]–[16]. For example, Yoshida et al. altered the schedule of restricted feeding only during the nocturnal period (i.e., a six-hour phase delay in feeding schedule) for four consecutive days, and found phase delays in clock-related gene expressions along with the timing of feeding [16]. In human studies, Goel et al. examined the association between dietary behavior and the circadian clock in patients with night eating syndrome [17], and found that the patients had a phase delay not only in the times of eating but also in the diurnal 24-h rhythms of physiological markers, such as melatonin, leptin, and insulin levels, compared to age and body mass index (BMI) matched healthy controls. Moreover, in our human studies in laboratory settings [18], [19], we found that later timing of breakfast and dinner caused a phase delay in the diurnal 24-h rhythm of cardiac autonomic nervous system activity assessed by heart rate variability (HRV). Therefore, there is a possibility that the timing of meal intake may be one of the causes for delayed circadian rhythm in rotating shift workers. However, it has remained unclear whether the timing of meal intake contributes to the modification of the rhythm in real-life situations. In addition, the relationship between phase angles of circadian and/or diurnal 24-h rhythms in HRV variables and energy intake at each meal, which is another property of dietary behavior, is also unclear.

HRV has been used extensively as a way to assess cardiac autonomic nervous system activity noninvasively in both laboratory and free-living settings [20]–[22]. We examined differences in the diurnal 24-h rhythms between shift workers and day workers by measuring the ambulatory HRV in real-life situations [13]. The results showed that shift workers had an abnormal phase angle between the diurnal 24-h variation in the cardiac autonomic nervous system activity and the sleep-wake cycle during the day shift. Therefore, the purpose of this study is to elucidate the association between dietary behavior and phase angles of diurnal 24-h variations in HRV variables among rotating shift workers while taking into account other behavioral factors such as the sleep-wake cycle and physical activity. Our hypothesis is that a later timing of meals contributes to delaying the phases of the variations in cardiac autonomic nervous system activity.

## Methods

### Participants

Nurses and caregivers working at a healthcare facility for the elderly in Choshi city, Chiba prefecture, Japan, participated in this cross-sectional study. The participants were 13 healthy Japanese (11 females and 2 males). None of the subjects took any over-the-counter or prescription medications for more than a month before the experiment. Inclusion criteria for study participants were: working for 40–46 hours a week (i.e., full-time worker) for at least 5 months consecutively in the current job and shift before the experiment; not obese (<30.0 kg/m^2^) [23]; no previous admission to hospital for cardiovascular disease or eating disorders. Their mean age and total duration of experience in rotating shift work were 38.6±10.4 (SD) yr (Female, 40.6±10.0 yr; Male, 27.5±0.7 yr) and 6.5±4.8 yr (Female, 6.6±5.2 yr; Male, 5.9±0.6 yr), respectively. The rotating 2-shift system was comprised of the day (09∶00–18∶00 h) and night (18∶00–09∶00 h) shifts. The average number of day and night shifts in the rotating shift during the previous month was 3 (range, 1–5) days and 1 (range, 0–2) night per week, respectively. Thus, the number of night shifts in the rotating shift was 4 or 5 nights per month. The measurements were conducted after 3.1±1.6 days of day shift or day off after a single night shift.

Each participant gave her/his written informed consent to participation, after the test protocol, aim, and prospective effect on health had been fully described. All the study procedures were reviewed and approved by the Institutional Review Board of the National Institute of Occupational Safety and Health, Japan.

### Protocol

Each participant was asked to undergo 24-h electrocardiograph (ECG) recording on the days of the day shift. The details of the study protocol are described in our previous study [13], which investigated the circadian characteristics among day and rotating shift workers. In brief, bipolar V5 lead ECG was recorded from the beginning of the day shift (09∶00 h) using a digital ambulatory ECG recorder (RAC3103, NIHON KOHDEN Corp, Tokyo, Japan). ECG signals were amplified and digitized at a sampling frequency of 250 Hz. Step counts were simultaneously recorded every two minutes using an electronic physical activity monitor (Lifecorder EX4, SUZUKEN Corp, Tokyo, Japan) [24]. The subjects wore their activity monitor attached to a waist belt. The subjects were instructed to record in a diary retiring and waking up times, start and end of times of work, and mealtimes. The total time in bed was calculated from recorded retiring and waking up times during off duty hours (18∶00–09∶00 h).

Food and beverages and the amounts of each consumed on the days of the experiment were also reported through dietary records. The subjects were asked to provide details of all food and beverages consumed (e.g., ingredients, quantities, cooking methods, and whether homemade or ready-made) and any nutritional supplements. At the end of the 24-h ECG recording, well-trained dietitians reviewed the dietary records for completeness and accuracy, then the total energy intake during the recording was calculated using the Standard Food Composition Table published by the Science and Technology Agency of Japan [25]. Meals taken between 05∶00 and 10∶00 h, 11∶00 and 15∶00 h, 17∶00 and 22∶00 h were defined as breakfast, lunch, and dinner, respectively [26]. Furthermore, the ratios of energy intake for breakfast, lunch, and dinner to total energy intake were calculated.

All the subjects worked the day shift or took a day off on the day before the day of the experiment, and their routine work schedules and contents during the days of the experiment were identical. The subjects were instructed that they should refrain from smoking, ingesting alcohol and caffeine, and engaging in prolonged and/or strenuous exercise, and should maintain habitual daily routines on the day before and the days of the experiment. The female subjects were all studied during the follicular phase of their menstrual cycles (i.e., between 5 and 12 days after the self-reported onset of menses).

### Time and frequency domain analysis of heart rate variability

The R-R interval (RRI) was derived from the ECG by using the first-derivative-based QRS detection method [27]. All RRI were scanned for extra or missing beats that could affect the results of the time and frequency domain analysis. The abnormal intervals were corrected by either the insertion (for missing beats) or the omission (for doubled or tripled beats) of beats. The number of beats corrected manually in this way was <0.5% of the total number of beats during the 24-h recording (about 100,000 beats).

Time (mean and SD of RRI) and frequency domain measures were calculated for approximately 10-min segments of HRV (600 beats) every 10 minutes. The HRV data of each segment were aligned sequentially to obtain equally spaced samples using the mean RRI [28]. After eliminating any linear trends by linear regression, coarse graining spectral analysis was used for 10-time-shifted subsets of 512 beats to break down the total power into regular periodic and fractal components [29], [30]. The total power of the periodic component (TOT: >0.04 Hz) and the integrated spectral power of periodic components in the low- (LF: 0.04–0.15 Hz) and high-frequency (HF: >0.15 Hz) ranges, the ratio of HF power to TOT (HF nu) were calculated, and also the ratio of LF power to HF power (LF/HF) [31]. HF nu and LF/HF have been used to assess cardiac vagal tone [20]–[22] and cardiac sympathovagal balance [29], [30], [32], respectively.

### Single cosinor analysis

Single cosinor analysis was performed on each subject’s data using the method of least squares regression to assess the timing and amplitude of the diurnal variation in selected HRV variables (mean and SD of RRI, HF nu, and LF/HF) or step counts for each subject after the HRV variables obtained or step counts totaled every 10 minutes were averaged for each 1-h bin for each subject [33], [34], respectively. In brief, the cosine function was represented as *y* = *M*+*A* cos (*ωt*−*φ*), where *M* is the mesor, *A* is the amplitude, *ω* = 2*π*/24, *t* is time, *φ* is the acrophase. According to this cosinor technique, two variables (amplitude and acrophase) characterized patterns associated with the diurnal 24-h rhythm in each of the HRV variables or step counts. The significance of the diurnal rhythm was tested by the zero-amplitude test [33], [35].

### Statistical analysis

We dichotomized the subjects’ data based on their breakfast time at its median into early and late eaters according to time since awakening to see the effects of the timing of breakfast after controlling for the sleep-wake cycle. An unpaired t-test or Mann-Whitney U test was used to test differences in variables between the groups. Simple correlation coefficients were calculated to explore associations between the timing and amount of each meal, the sleep-wake cycle, and physical activity and phase angle of 24-h rhythm in each HRV variable. Multivariable linear regressions were used to explore the relative contribution of the timing and the amount of each meal and the total energy intake to the phase angle of each HRV variable. In these analyses, the acrophases of HRV variables and meal times were expressed in time since awakening. A p value of less than 0.05 was considered as statistically significant using a two-tailed test. Corrections for multiple tests in multivariable linear regressions were performed using the Benjamini-Hochberg procedure [36]. The Benjamini-Hochberg test controls for the false discovery rate (FDR) using sequential modified Bonferroni correction for multiple hypothesis testing. An initial FDR threshold was 0.05. All statistical analyses were performed with an SPSS software package (IBM SPSS 20.0 for Windows, SPSS Japan, Tokyo, Japan).

## Results

The mean age, height, weight, and BMI of the subjects were 38.6 (range, 27–53) yr, 161.7 (range, 154–178) cm, 59.4 (range, 44–79) kg, and 22.6 (range, 18.6–28.0) kg/m^2^, respectively. On the experimental day, means of daily energy intake and the ratios of energy intake at breakfast, lunch, dinner, and snacks were 1962±274 (SD) kcal, 16.9±8.1%, 30.9±5.7%, 47.2±10.9%, and 4.9±5.8%, respectively. The means of the timings of breakfast, lunch, dinner, waking up, and retiring were 7∶15±0∶35 h, 12∶34±0∶34 h, 19∶33±0∶56 h, 6∶30±0∶51 h, and 23∶32±1∶29 h, respectively. All snacks were taken between breakfast and lunch, or lunch and dinner.

**Figure 1:**
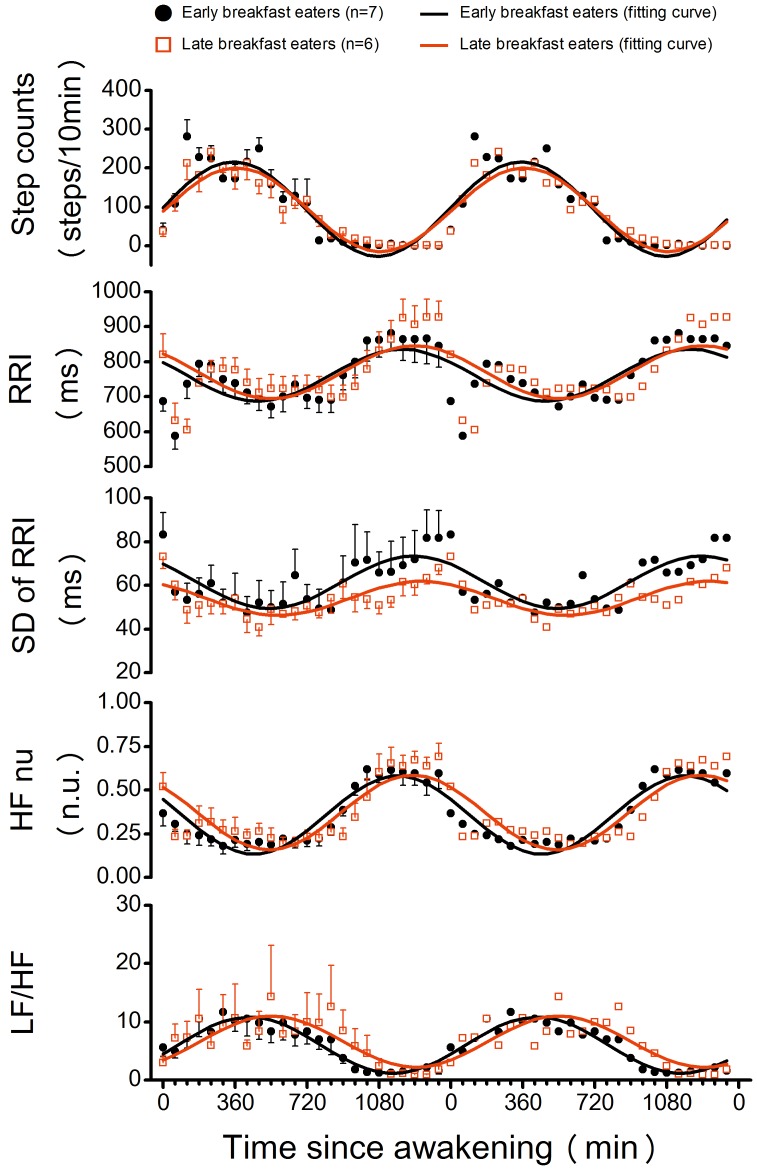
Ensemble-averaged step counts and heart rate variability variables during the day shift over 24 h. Values are mean ± SE. Ensemble-averaged fitted curves by using single cosinor analysis for early eaters (black line) and late eaters (red line) depending on breakfast timings according to time since awakening are also depicted. Data are double-plotted to better visualize rhythmicity. HF nu, the ratio of high frequency (HF: >0.15 Hz) power to total power (>0.04 Hz); LF/HF, the ratio of low frequency (LF: 0.04–0.15 Hz) power to HF power.

Figure 1 depicts double plots of ensemble-averaged 10-min step counts and HRV variables over 24 h for early breakfast eaters and late breakfast eaters. The means of breakfast time expressed in time since awakening were 15±13 min and 79±22 min for the early and late breakfast eaters, respectively (Table 1). Step counts and HRV variables for each 1-h bin were averaged across all subjects. Ensemble-averaged fitted curves calculated by cosinor analysis (averaged across all subjects) are also shown for each group. When data was compared based on time since awakening, acrophases of 24-h period variations for HF nu and LF/HF were significantly (p<0.05) delayed in the late breakfast eaters compared to those in the early breakfast eaters (Table 1). The acrophases of 24-h period variation for step counts and retiring time, as well as awakening time, did not differ (p>0.05) between the groups (Table 1).

**Table 1:** General characteristic of subjects dichotomized into early eaters and late eaters depending on breakfast timing (median: 30 min).

		Early eaters	Late eaters	p values
		n = 7	n = 6	
Age†	(yr)	36.9±11.1	40.7±10.1	0.534
Experience of shift work‡	(yr)	6.9±5.8	6.0±3.6	0.830
Height†	(cm)	162.6±9.9	160.6±5.1	0.674
Weight†	(kg)	57.1±10.9	62.0±9.0	0.410
BMI†	(kg/m^2^)	21.5±2.3	24.0±2.5	0.085
Step counts†	(steps/day)	11928±1925	13212±2704	0.398
Female/Male§		5/2	6/0	0.462
Energy intake				
Total†	(kcal)	1797±180	2155±242*	0.011
Breakfast E%^a^ ‡	(%)	17.2±9.8	16.5±6.5	0.475
Lunch E%^a^ †	(%)	32.1±5.6	29.6±6.1	0.463
Dinner E%^a^ †	(%)	45.6±10.8	49.0±11.8	0.597
Sleeping hour†	(min)	435±41	395±44	0.113
Timing¶				
Retiring†	(min)	1005±41	1045±44	0.113
Breakfast‡	(min)	15±13	79±22*	<0.001
Lunch‡	(min)	343±21	388±60	0.150
Dinner‡	(min)	743±71	830±54*	0.032
Acrophase				
Step counts†	(min)	353±54	361±63	0.803
RRI Mean†	(min)	1195±64	1289±158	0.171
SD‡	(min)	1315±167	1317±135	1.000
HF nu†	(min)	1173±53	1260±56*	0.015
LF/HF†	(min)	419±64	528±78*	0.018

Values are shown as mean ± SD.

BMI, body mass index; RRI, R-R interval; HF nu, the ratio of high frequency (HF: >0.15 Hz) power to total power (>0.04 Hz); LF/HF, the ratio of low frequency (LF: 0.04–0.15 Hz) power to HF power.

*Significantly different between the groups (p<0.05).

† Non-paired t test;

‡ Mann-Whitney U test;

§ Chi-squared test.

¶ Time since awakening.

a The ratio of energy intake to total energy intake (E%).

**Figure 2:**
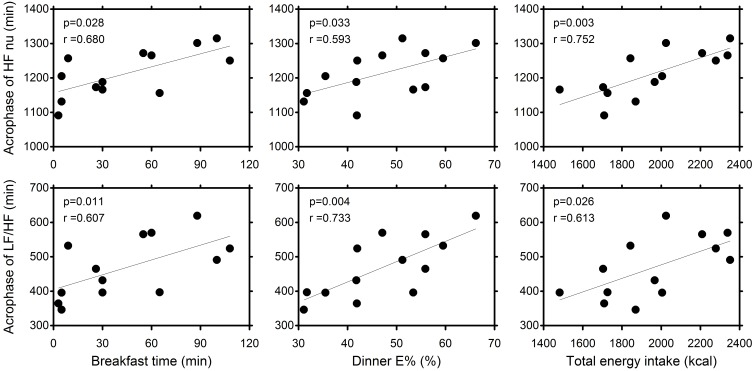
The relations between breakfast time, the ratio of energy intake at dinner to total energy intake (E%), and total energy intake and the acrophases of HF nu and LF/HF according to data based on time since awakening. Pearson correlation coefficients are presented.

We next explored the correlation between the timing and amount of each meal, retiring time, and the timing and amount of physical activity and the acrophases of HF nu and LF/HF (Table 2). Data based on time since awakening showed that the timing of breakfast, the ratio of energy intake at dinner, and total energy intake were significantly (p<0.05) correlated to the acrophases of HF nu and LF/HF (Figure 2), while the time of retiring was not (Table 2). The ratio of energy intake at lunch was significantly (p<0.05) correlated only to the acrophases of HF nu. No variables in physical activity were correlated to the acrophases of HF nu or LF/HF.

**Table 2:** Pearson correlation coefficients for acrophases of HRV variables.

	Acrophase of HF nu¶	Acrophase of LF/HF¶
Age	0.250	0.109
Experience of shift work	−0.274	−0.139
Height	−0.344	−0.286
Weight	−0.121	−0.171
BMI	0.119	0.000
Step counts	0.190	0.223
Energy intake		
Total	0.752*	0.613*
Breakfast E%	−0.294	−0.422
Lunch E%	−0.607*	−0.542
Dinner E%	0.593*	0.733*
Sleeping hour	−0.284	−0.284
Timing¶		
Acrophase of step counts	0.072	0.110
Retiring	0.380	0.284
Breakfast	0.680*	0.607*
Lunch	0.376	0.241
Dinner	0.378	0.291

*p<0.05.

¶ Time since awakening.

Multivariable linear regression analysis was then carried out to investigate the effects of the timing and amount of each meal and total energy intake on the acrophases of HF nu and LF/HF separately. Sex and BMI were statistically controlled for because both the male subjects were early breakfast eaters (Table 1) and BMI was different between the late and early breakfast eaters at a trend level (Table 1). The timing of breakfast had a significant effect (p<0.05; significant following FDR correction) on the acrophases of HF nu (β = 0.804) and LF/HF (β = 0.827) after controlling for sex and BMI (Table 3). There were also significant effects (p<0.05; significant following FDR correction) of the ratio of energy intake at dinner on the acrophases of HF nu (β = 0.789) and LF/HF (β = 0.916). Total energy intake had a significant effect (p<0.05; significant following FDR correction) on the acrophases of HF nu (β = 1.072) and LF/HF (β = 0.987).

**Table 3:** Multivariable linear regression analysis for the acrophases of HRV variables.

		Unstandardized		Standardized		FDR	
	Independent variables	coefficients		coefficients	p values	threshold†	R^2^
		B	SE	β			
Acrophase of HF nu (min)¶							
Model 1	Timing of breakfast (min)¶	1.490	0.529	0.804	**0.020** *	0.031	0.557
Model 2	Lunch E% (%)	−6.469	3.297	−0.536	0.081	0.044	0.416
Model 3	Dinner E% (%)	4.973	1.484	0.789	**0.009** *	0.025	0.629
Model 4	Total energy intake (kcal)	0.270	0.054	1.072	**0.001** *	0.006	0.782
Acrophase of LF/HF (min)¶							
Model 1	Timing of breakfast (min)¶	1.955	0.704	0.827	**0.022** *	0.038	0.517
Model 2	Lunch E% (%)	−7.904	4.497	−0.514	0.113	0.050	0.332
Model 3	Dinner E% (%)	7.361	1.626	0.916	**0.001** *	0.013	0.726
Model 4	Total energy intake (kcal)	0.317	0.090	0.987	**0.006** *	0.019	0.624

Model 1–4, adjusted by sex and BMI.

*p<0.05.

† P-value < false discovery rate (FDR) threshold is significant. P-values which remain significant following FDR corrections for multiple comparisons are in bold.

¶ Time since awakening.

## Discussion

In this study, we have investigated the associations between dietary behavior and phase angle of diurnal 24-h rhythm in cardiac autonomic nervous system activity among rotating shift workers in real-life situations. To standardize the effect of the sleep-wake cycle and 24-h rhythm of physical activity, the time series data for step counts and HRV variables were aligned based on the waking time. As a result, transient changes in RRI immediately after awakening were also aligned, as shown in Figure 1. HRV variables such as HF nu and LF/HF for each subject still show 24-h rhythms. For example, LF/HF has a peak during the daytime and a trough during the night time, which is consistent with diurnal variations in markers of sympathetic nervous system activity observed in previous studies [37], [38]. The results also show that the acrophases of HF nu and LF/HF were significantly delayed in late breakfast eaters according to data based on time since awakening. Multivariable regression analysis revealed that there was a significant effect of the timing of breakfast on the acrophases of HF nu and LF/HF (Table 3). In addition, there were significant effects of the ratio of energy intake at dinner and total energy intake on the acrophases of HF nu and LF/HF (Table 3). These results did not change when multivariate regression analysis was conducted after controlling for sex, BMI, and age, or in the case of data from women, after controlling for BMI, except for the relationship between the timing of breakfast and the acrophase of LF/HF (p = 0.047; not significant following FDR correction).

Our previous studies, which fixed the sleep-wake cycle (00∶00–06∶00 h) and restricted physical activity, showed that delayed or advanced feeding schedules over 14 days resulted, respectively, in delayed or advanced phase angles of diurnal 24-h rhythms of cardiac autonomic nervous system activity [18], [19]. In this study, there was a correlation between the timing of breakfast and the phase angle of diurnal 24-h rhythms in HF nu and LF/HF for data based on time since awakening. These data indicate the possibility that the timing of breakfast is related to phase angles of diurnal 24-h rhythms of cardiac autonomic nervous system activity independently of the effects on the rhythms of other zeitgebers in humans (i.e., light and physical activity). However, the effects of the timing of breakfast on the phase angles of cardiac autonomic nervous system activity might be overestimated, because we were not able to control for the effects of other dietary behaviors or factors with the sample size of this study.

Our results show that the ratio of energy intake at dinner was also associated with the acrophases of HF nu and LF/HF. Kuroda et al. [39] revealed that the amount of a meal might determine the response of phase shifting of the circadian clock in peripheral tissues. Although we were not able to examine the effects of the amount of each meal on the acrophases independently of the timing of breakfast with the sample size of this study, it should be examined whether it is one of the influential factors for phase shift in the circadian clock in real-life situations.

Considering the fact that heart rate in SCN-intact, but not SCN-lesioned rats had a clear circadian rhythm and that the diurnal pattern of the HRV could be mediated through projections from the SCN to the autonomic subdivision of the paraventricular nucleus of the hypothalamus [40], our results also suggest that shift workers who had breakfast at a later time after awakening may have a tendency toward more phase delay of the clock in the SCN, and may have more severe misalignment between the clock and the sleep-wake cycle. The strongest stimuli for entraining the clock to the sleep-wake cycle and/or external day-night cycle (i.e., phase resetting) is the light. Recently, it has been reported that non-photic factors such as food intake, can also have an effect on the phase of the clock [14], [41]. Although, the physiological mechanisms for the phase resetting effects of the timing and amount of food intake are unclear, one of the candidates is the humoral factor. For example, a previous study showed that ghrelin-secreting oxyntic cells of the stomach are oscillators entrained by food, and that they produce a systemic output signal that could set the phase of other oscillators in the body [42]. Ghrelin receptors are widely distributed in both brain and peripheral sites, and the SCN responds to direct ghrelin application [43]. Further study of the physiological mechanisms of the association between food intake and the circadian clock in humans is needed. Our study shows that the effects of the amount of the meals at lunch and dinner, which were not correlated (r = −0.308, p = 0.306) with each other, were opposite in direction (i.e., advance and delay, respectively) (Table 3), indicating that the magnitude and direction of phase response might depend on both the timing and the amount of food intake, as is the case in the phase response induced by light in humans.

A recent previous study showed that the circadian clock regulates hunger and appetite independently of the sleep-wake cycle and the fasting-feeding rhythm in humans in a laboratory setting [44], indicating that there is a possibility that the timing of breakfast might be changed by the phase of the circadian clock. However, it should be noted that all data in our study were obtained on days of day shift, which could be a social factor and restrict temporal patterns of behaviors of the subjects. All the subjects lived their life as usual on days of the day shift during the days of the experiment. For example, the timing of awakening, which could be controlled partly by the circadian clock, did not have significant relationships with the acrophases of HF nu and LF/HF for data based on the clock time. Thus, it might be speculated that the effects of the circadian clock on the timing of breakfast are smaller than might be expected under conditions without social restrictions.

Night work causes a phase shift in the circadian clock [11], [45]. Our previous study indicated that the phase angle of 24-h rhythm in cardiac autonomic nervous system activity among shift workers who engage in night shifts 1 or 2 times per week was significantly delayed compared with that in day workers even on the days of the day shift, while the sleep-wake cycle was not [13]. For day workers in that study, acrophases of 24-h period variations for HF nu and LF/HF based on time since awakening were 1172±74 min and 412±84 min, respectively, which were comparable to data for the early breakfast eaters in the present study. Thus, this study indicates that the delay and the misalignment may be partly associated with dietary behavior. Recent laboratory studies have indicated that an abnormal phase angle between the circadian clock and the sleep-wake cycle (approximately 4.0–6.0 h) can have adverse effects on metabolism (e.g., glucose, insulin, triglyceride, and leptin) [46]–[48] and might cause health problems such as cardiovascular disease and metabolic disorders [7], [48]. Although, it is possible for the phase angle between the circadian clock and the sleep-wake cycle of shift workers to return to normal after keeping to a regular 7.5- to 8.0-h nocturnal sleep pattern for 8 days [37], a quick improvement in the abnormal phase angle during the day shift may be important in preventing health problems in rotating shift workers. Future study is needed to examine whether dietary behavior is one of the zeitgebers, and whether controlling dietary behavior improves the misalignment rapidly and prevents health problems in shift workers. Moreover, it might be important to reveal factors that determine the length of time from awakening to breakfast. Sex and age were not associated with the length of time in our sample.

There are some limitations to our study. First, the effects of behavioral factors which can modify the circadian pattern of cardiac autonomic nervous system activity could not be completely standardized in our free-living conditions. Second, self-reported data on the onset of meals and the sleep-wake cycle could be inherently biased. Third, dietary content was not considered. Fourth, other zeitgebers and confounders which were not measured in this study could not be considered. The effects of dietary behavior on the circadian clock in shift workers need to be further investigated using a larger sample size and more sensitive measures of circadian phase, such as the acrophase of 24-h melatonin concentration or the dim light melatonin onset test.

In conclusion, the timing of breakfast, the ratio of energy intake at dinner, and total energy intake were associated with the phase angle for diurnal 24-h variations of cardiac autonomic nervous system activity on days of the day shift in rotating shift workers independently of the sleep-wake cycle and physical activity. Therefore, the phase angle between the circadian variation in cardiac autonomic nervous system activity and the sleep-wake cycle may be associated partly with dietary behavior in rotating shift workers. Future studies should examine causality between dietary behavior and the circadian rhythm in rotating shift workers to find how to adjust the circadian clock to the sleep-wake cycle quickly after the night shift. This may be significant in preventing health problems in shift workers.
